# Supplementary material for: The Association between Term Chorioamnionitis during Labor and Long-Term Infectious Morbidity of the Offspring
Source: J Clin Med. 2024 Jan 31;13(3):814. doi: 10.3390/jcm13030814 (PMC10856245; doi:10.3390/jcm13030814)
Supplement: Supplementary file 1 [file jcm-13-00814-s001.zip › jcm-2816486-SI.pdf]

**Table S1:** ICD-9 Codes for pediatric infectious morbidity

| Group | Diag. code | Diagnosis description |
|---|---|---|
| **Bacteremia / Septicemia** | 7907 | BACTEREMIA |
| | 99591 | SEPSIS |
| | 99592 | SEVERE SEPSIS |
| | 99592 | SYSTEMIC INFLAMMATORY RESPONSE SYNDROME DUE TO INFECTIOUS PROCESS WITH ORGAN DYSFUNCTION |
| **Bacterial infections** | 0414 | ESCHERICHIA COLI (E. COLI), UNSP. SITE |
| | 0414 | ESCHERICHIA COLI (E. COLI), UNSP. SITE (ADDITIONAL CODE) |
| | 0414 | ESCHERICHIA COLI(E. COLI),CONDITI.CLASSIF.ELSEWHERE,UNSP.SITE |
| | 0413 | FRIEDLANDER'S BACILLUS, UNSP. SITE |
| | 0413 | FRIEDLANDER'S BACILLUS;CONDITION CLASSIF.ELSEWHERE,UNSP.SITE |
| | 0415 | HEMOPHILUS INFLUENZAE IN CONDIT.CLASSIF.ELSEWHERE,UNSP.SITE |
| | 0415 | HEMOPHILUS INFLUENZAE, UNSP. SITE (H.INFLUENZAE) |
| | 0413 | KLEBSIELLA PNEUMONIAE |
| | 0369 | MENINGOCOCCAL INFECTION, UNSPECIFIED |
| | 04112 | METHICILLIN RESISTANT STAPHYLOCOCCUS AUREUS |
| | 04111 | METHICILLIN SUSCEPTIBLE STAPHYLOCOCCUS AUREUS |
| | 04185 | OTHER GRAM-NEGATIVE ORGANISMS INFECTION |
| | 03689 | OTHER SPECIFIED MENINGOCOCCAL INFECTIONS |
| | 04119 | OTHER STAPHYLOCOCCUS INFECTION |
| | 04109 | OTHER STREPTOCOCCUS INFEC. |
| | 0412 | PNEUMOCOCCUS INFECT.IN CONDITION CLASSIF.ELSEWHERE;UNSP.SITE |
| | 0412 | PNEUMOCOCCUS INFECTION, UNSP. SITE |
| | 0416 | PROTEUS (MIRABILIS,MORGANII), UNSP. SITE |
| | 0416 | PROTEUS(MIRABILIS,MORGANII)CONDIT,CLASSIF.ELSEWHERE,UNSP.SITE |
| | 0417 | PSEUDOMONAS INFEC., UNSP. SITE |
| | 0417 | PSEUDOMONAS INFECT.IN CONDIT.CLASSIF.ELSEWHERE,UNSPEC.SITE |
| | 04111 | STAPHYLOCOCCUS AUREUS INFEC. |
| | 04111 | STAPHYLOCOCCUS AUREUS INFEC. (ADDITIONAL CODE) |
| | 0411 | STAPHYLOCOCCUS INFECT.IN CONDIT.CLASSIF.ELSEWHERE,UNSP.SITE |
| | 04110 | STAPHYLOCOCCUS INFECTION, UNSP. |
| | 0411 | STAPHYLOCOCCUS INFECTION, UNSP. SITE |
| | 04101 | STREPTOCOCCUS INFEC., GROUP A |
| | 04101 | STREPTOCOCCUS INFEC., GROUP A (ADDITIONAL CODE) |
| | 04102 | STREPTOCOCCUS INFEC., GROUP B |
| | 04103 | STREPTOCOCCUS INFEC., GROUP C |
| | 04104 | STREPTOCOCCUS INFEC., GROUP D (ENTEROCOCCUS) |
| | 04105 | STREPTOCOCCUS INFEC., GROUP G |
| | 04100 | STREPTOCOCCUS INFEC., UNSP. |
| | 0410 | STREPTOCOCCUS INFECT.IN CONDITION CLASSIF.ELSEWHERE;UNSP.SITE |
| **Blood-borne virus infections** | 042 | HUMAN IMMUNODEFIC. VIRUS (HIV) DIS. /AIDS |
| | 07959 | OTHER SPECIFIED RETROVIRUS |
| | 07950 | RETROVIRUS, UNSP.,UNSP. SITE |

| | | |
|---|---|---|
| | 0709 | UNSP. VIRAL HEPATITIS WITHOUT HEPATIC COMA |
| | 0709 | UNSPECIFIED VIRAL HEPATITIS WITHOUT MENTION OF HEPATIC COMA |
| | 07030 | VIRAL HEPATITIS B WITHOUT HEPATIC COMA & HEPATITIS DELTA -92 |
| | 07030 | VIRAL HEPATITIS B WITHOUT HEPATIC COMA,AC/UNSP.WITHOUT HEP. DELTA |
| | 07032 | VIRAL HEPATITS B WITHOUT HEPATIC COMA,CHR. WITHOUT HEPATITIS DELT |
| | V08 | ASYMPTOMATIC H.I.V INFECTION STATUS |
| **Cardiovascular infections** | 4211 | ACUTE + SUBACUTE INFEC.ENDOCARDITIS IN DIS.CLASS.ELSEWHERE |
| | 4210 | ACUTE AND SUBACUTE BACTERIAL ENDOCARDITIS |
| | 42290 | ACUTE MYOCARDITIS, UNSPECIFIED |
| | 42090 | ACUTE PERICARDITIS, UNSPECIFIED |
| | 42291 | IDIOPATHIC MYOCARDITIS |
| | 4290 | MYOCARDITIS, UNSPECIFIED |
| | 42099 | OTHER ACUTE PERICARDITIS |
| | 42292 | SEPTIC MYOCARDITIS |
| | 41512 | SEPTIC PULMONARY EMBOLISM |
| **CNS infections** | 320 | BACTERIAL MENINGITIS |
| | 3200 | HEMOPHILUS MENINGITIS |
| | 3240 | INTRACRANIAL ABSCESS |
| | 3249 | INTRACRANIAL AND INTRASPINAL ABSCESS OF UNSPECIFIED SITE |
| | 3241 | INTRASPINAL ABSCESS |
| | 326 | LATE EFFECTS OF INTRACRANIAL ABSCESS OR PYOGENIC INFECTION |
| | 32082 | MENINGITIS DUE TO GRAM-NEGATIVE |
| | 3208 | MENINGITIS DUE TO OTHER SPECIFIED BACTERIA |
| | 32089 | MENINGITIS DUE TO OTHER SPECIFIED BACTERIA |
| | 3209 | MENINGITIS DUE TO UNSPECIFIED BACTERIUM |
| | 3207 | MENINGITIS IN OTHER BACTERIAL DISEASES CLASSIFIED ELSEWHERE |
| | 325 | PHLEBITIS AND THROMBOPHLEBITIS OF INTRACRANIAL VENOUS SINUSES |
| | 3201 | PNEUMOCOCCAL MENINGITIS |
| | 3203 | STAPHYLOCOCCAL MENINGITIS |
| | 3202 | STREPTOCOCCAL MENINGITIS |
| | 05472 | HERPES SIMPLEX MENINGITIS |
| | 0543 | HERPETIC MENINGOENCEPHALITIS |
| | 32361 | INFECTIOUS ACUTE DISSEMINATED ENCEPHALOMYELITIS (ADEM) |
| | 0470 | MENINGITIS DUE TO COXSACKIE VIRUS |
| | 0471 | MENINGITIS DUE TO ECHO VIRUS |
| | 0360 | MENINGOCOCCAL MENINGITIS |
| | 0362 | MENINGOCOCCEMIA |
| | 0491 | NON-ARTHOPOD-BORNE MENINGITIS DUE TO ADENOVIRUS |
| | 048 | OTHER ENTEROVIRUS DISEASES OF CENTRAL NERVOUS SYSTEM |
| | 05829 | OTHER HUMAN HERPESVIRUS ENCEPHALITIS |
| | 0478 | OTHER SPECIFIED VIRAL MENINGITIS |
| | 3236 | POSTINFECTIOUS ENCEPHALITIS |
| | 0630 | RUSSIAN SPRING-SUMMER (TAIGA) ENCEPHALITIS |

| | | |
|---|---|---|
| | 0499 | UNSP.NON-ARTHROPOD-BORNE VIRAL DIS.OF CENTRAL NERVOUS SYSTEM |
| | 0479 | UNSPECIFIED VIRAL MENINGITIS |
| | 06641 | WEST NILE FEVER WITH ENCEPHALITIS |
| **ENT infections** | 38200 | AC.SUPPURAT.OTITIS MEDIA WITHOUT SPONTAN.RUPTURE OF EARDRUM |
| | 38104 | ACUTE ALLERGIC SEROUS OTITIS MEDIA |
| | 38300 | ACUTE MASTOIDITIS WITHOUT COMPLICATIONS |
| | 38400 | ACUTE MYRINGITIS, UNSPECIFIED |
| | 38100 | ACUTE NONSUPPURATIVE OTITIS MEDIA, UNSPECIFIED |
| | 38001 | ACUTE PERICHONDRITIS OF PINNA |
| | 38101 | ACUTE SEROUS OTITIS MEDIA |
| | 38201 | ACUTE SUPPURAT.OTITIS MEDIA WITH SPONTAN.RUPTURE OF EARDRUM |
| | 3820 | ACUTE SUPPURATIVE OTITIS MEDIA |
| | 38401 | BULLOUS MYRINGITIS |
| | 11282 | CANDIDAL OTITIS EXTERNA |
| | 38531 | CHOLESTEATOMA OF ATTIC |
| | 38532 | CHOLESTEATOMA OF MIDDLE EAR |
| | 38530 | CHOLESTEATOMA, UNSPECIFIED |
| | 38003 | CHONDRITIS OF PINNA |
| | 3831 | CHRONIC MASTOIDITIS |
| | 38015 | CHRONIC MYCOTIC OTITIS EXTERNA |
| | 38002 | CHRONIC PERICHONDRITIS OF PINNA |
| | 38110 | CHRONIC SEROUS OTITIS MEDIA, SIMPLE OR UNSPECIFIED |
| | 3821 | CHRONIC TUBOTYMPANIC SUPPURATIVE OTITIS MEDIA |
| | 0740 | HERPANGINA |
| | 38010 | INFECTIVE OTITIS EXTERNA, UNSPECIFIED |
| | 3814 | NONSUPPURATIVE OTITIS MEDIA, NOT SPECIFIED AS ACUTE OR CHRONIC |
| | 38022 | OTHER ACUTE OTITIS EXTERNA |
| | 3813 | OTHER AND UNSPECIFIED CHRONIC NONSUPPURATIVE OTITIS MEDIA |
| | 38129 | OTHER CHRONIC MUCOID OTITIS MEDIA |
| | 38023 | OTHER CHRONIC OTITIS EXTERNA |
| | 38119 | OTHER CHRONIC SEROUS OTITIS MEDIA |
| | 38330 | POSTMASTOIDECTOMY COMPLICATION, UNSPECIFIED |
| | 38301 | SUBPERIOSTEAL ABSCESS OF MASTOID |
| | 382 | SUPPURATIVE AND UNSPECIFIED OTITIS MEDIA |
| | 3823 | UNSPECIFIED CHRONIC SUPPURATIVE OTITIS MEDIA |
| | 3839 | UNSPECIFIED MASTOIDITIS |
| | 3829 | UNSPECIFIED OTITIS MEDIA |
| | 3824 | UNSPECIFIED SUPPURATIVE OTITIS MEDIA |
| | 101 | VINCENT'S ANGINA |
| **Fungal infections** | 1173 | ASPERGILLOSIS |
| | 1160 | BLASTOMYCOSIS |
| | 11281 | CANDIDAL ENDOCARDITIS |
| | 11284 | CANDIDAL ESOPHAGITIS |

| | | |
|---|---|---|
| | 1124 | CANDIDIASIS OF LUNG |
| | 1129 | CANDIDIASIS OF UNSPECIFIED SITE |
| | 1175 | CRYPTOCOCCOSIS |
| | 1228 | ECHINOCOCCOSIS, UNSPECIFIED, OF LIVER |
| | 1225 | ECHINOCOCCUS MULTILOCULARIS INFECTION OF LIVER |
| | 1179 | OTHER AND UNSPECIFIED MYCOSES |
| | 11289 | OTHER CANDIDIASIS OF OTHER SPECIFIED SITES |
| | 1209 | SCHISTOSOMIASIS, UNSPECIFIED |
| | 1177 | ZYGOMYCOSIS (PHYCOMYCOSIS OR MUCORMYCOSIS) |
| **GI infections** | 0069 | AMEBIASIS, UNSPECIFIED |
| | 0068 | AMEBIC INFECTION OF OTHER SITES |
| | 1270 | ASCARIASIS |
| | 0085 | BACTERIAL ENTERITIS, UNSPECIFIED |
| | 0070 | BALANTIDIASIS |
| | 0051 | BOTULISM |
| | 0091 | COLITIS,ENTERITIS,GASTROENTERITIS OF PRESUMED INF. ORIGIN |
| | 0074 | CRYPTOSPORIDIOSIS |
| | 0075 | CYCLOSPORIASIS |
| | 1229 | ECHINOCOCCOSIS, OTHER AND UNSPECIFIED |
| | 00862 | ENTERITIS DUE TO ADENOVIRUS |
| | 00865 | ENTERITIS DUE TO CALICIVIRUS |
| | 00867 | ENTERITIS DUE TO ENTEROVIRUS, N.E.C. |
| | 00861 | ENTERITIS DUE TO ROTAVIRUS |
| | 1274 | ENTEROBIASIS |
| | 0059 | FOOD POISONING, UNSPECIFIED |
| | 0071 | GIARDIASIS |
| | 04186 | HELICOBACTER PYLORI (H. PYLORI) INFECTION |
| | 1289 | HELMINTH INFECTION, UNSPECIFIED |
| | 1236 | HYMENOLEPIASIS |
| | 0090 | INFECTIOUS COLITIS, ENTERITIS, & GASTROENTERITIS |
| | 0090 | INFECTIOUS COLITIS, ENTERITIS, AND GASTROENTERITIS |
| | 0092 | INFECTIOUS DIARRHEA |
| | 1279 | INTESTINAL HELMINTHIASIS, UNSPECIFIED |
| | 0082 | INTESTINAL INFEC. DUE TO AEROBACTER AEROGENES |
| | 00843 | INTESTINAL INFEC. DUE TO CAMPYLOBACTER |
| | 00842 | INTESTINAL INFEC. DUE TO PSEUDOMONAS |
| | 00841 | INTESTINAL INFEC. DUE TO STAPHYLOCOCCUS |
| | 0088 | INTESTINAL INFECTION DUE TO OTHER ORGANISM,NOT ELSEW.CLASS. |
| | 0084 | INTESTINAL INFECTION DUE TO OTHER SPECIFIED BACTERIA |
| | 008 | INTESTINAL INFECTIONS DUE TO OTHER ORGANISMS |
| | 129 | INTESTINAL PARASITISM, UNSPECIFIED |
| | 0073 | INTESTINAL TRICHOMONIASIS |
| | 0270 | LISTERIOSIS |
| | 1278 | MIXED INTESTINAL HELMINTHIASIS |
| | 00329 | OTHER LOCALIZED SALMONELLA INFECTIONS |

| | | |
|---|---|---|
| | 0078 | OTHER SPECIFIED PROTOZOAL INTESTINAL DISEASES |
| | 0038 | OTHER SPECIFIED SALMONELLA INFECTIONS |
| | 0048 | OTHER SPECIFIED SHIGELLA INFECTIONS |
| | 00869 | OTHER VIRAL ENTERITIS |
| | 0022 | PARATYPHOID FEVER B |
| | 0023 | PARATYPHOID FEVER C |
| | 00323 | SALMONELLA ARTHRITIS |
| | 0030 | SALMONELLA GASTROENTERITIS |
| | 0039 | SALMONELLA INFECTION, UNSPECIFIED |
| | 00321 | SALMONELLA MENINGITIS |
| | 0031 | SALMONELLA SEPTICEMIA |
| | 0042 | SHIGELLA BOYDII |
| | 0040 | SHIGELLA DYSENTERIAE |
| | 0041 | SHIGELLA FLEXNERI |
| | 0043 | SHIGELLA SONNEI |
| | 0049 | SHIGELLOSIS, UNSPECIFIED |
| | 1369 | UNSP. INFECTIOUS & PARASITIC DISEASES |
| | 1369 | UNSPECIFIED INFECTIOUS AND PARASITIC DISEASES |
| | 0079 | UNSPECIFIED PROTOZOAL INTESTINAL DISEASE |
| **Gynecological infections** | 6163 | ABSCESS OF BARTHOLIN'S GLAND |
| | 64663 | ANTEPARTUM INFECTIONS OF GENITOURINARY TRACT |
| | 1121 | CANDIDIASIS OF VULVA AND VAGINA |
| | 6162 | CYST OF BARTHOLIN'S GLAND |
| | 64662 | INFECT.OF GENITOURINARY TR.IN PREGN.,WITH DELIV.,WITH POSTP.COMPL. |
| | 64660 | INFECTIONS OF GENITOURINARY TRACT IN PREGN.,UNSP.EPISODE |
| | 6164 | OTHER ABSCESS OF VULVA |
| | 64664 | POSTPARTUM INFECTIONS OF GENITOURINARY TRACT |
| | 61610 | VAGINITIS AND VULVOVAGINITIS, UNSPECIFIED |
| **Invasive bacterial infections** | 0388 3 | ACITINOBACTER SEPTICEMIA |
| | 0388 1 | CANDIDA SEPTICEMIA |
| | 0388 2 | ENTEROCOCCUS SEPTICEMIA |
| | 038491 | KLEBSIELLA SEPTICEMIA |
| | 03812 | METHICILLIN RESISTANT STAPHYLOCOCCUS AUREUS SEPTICEMIA |
| | 03811 | METHICILLIN SUSCEPTIBLE STAPHYLOCOCCUS AUREUS SEPTICEMIA |
| | 03849 | OTHER SEPTICEMIA DUE TO GRAM-NEGATIVE ORGANISMS |
| | 0388 | OTHER SPECIFIED SEPTICEMIAS |
| | 03819 | OTHER STAPHYLOCOCCAL SEPTICEMIA |
| | 0382 | PNEUMOCOCCAL SEPTICEMIA |
| | 0383 | SEPTICEMIA DUE TO ANAEROBES |
| | 03842 | SEPTICEMIA DUE TO ESCHERICHIA COLI (E. COLI) |
| | 03840 | SEPTICEMIA DUE TO GRAM-NEGATIVE ORGANISM, UNSPECIFIED |
| | 03841 | SEPTICEMIA DUE TO HEMOPHILUS INFLUENZAE (H. INFLUENZAE) |
| | 03843 | SEPTICEMIA DUE TO PSEUDOMONAS |
| | 0381 | STAPHYLOCOCCAL SEPTICEMIA |

| | | |
|---|---|---|
| | 03810 | STAPHYLOCOCCAL SPETICEMIA, UNSP. |
| | 03811 | STAPHYLOCOCCUS AUREUS SEPTICEMIA |
| | 0380 | STREPTOCOCCAL SEPTICEMIA |
| | 0389 | UNSPECIFIED SEPTICEMIA |
| **Neonatal infections** | 7775 | NECROTIZING ENTEROCOLITIS IN FETUS OR NEWBORN |
| | 77750 | NECROTIZING ENTEROCOLITIS IN NEWBORN, UNSPECIFIED |
| | 77753 | STAGE III NECROTIZING ENTEROCOLITIS IN NEWBORN |
| | 77183 | BACTEREMIA OF NEWBORN |
| | 7711 | CONGENITAL CYTOMEGALOVIRUS INFECTION |
| | 7700 | CONGENITAL PNEUMONIA |
| | 771 | INFECTIONS SPECIFIC TO THE PERINATAL PERIOD |
| | 7717 | NEONATAL CANDIDA INFECTION |
| | 7716 | NEONATAL CONJUNCTIVITIS AND DACRYOCYSTITIS |
| | 7718 2 | NEONATAL SEPSIS WORK UP |
| | 7718 1 | NEONATAL URINARY TRACT INFECTION |
| | 7712 | OTHER CONGENITAL INFECTIONS SPECIFIC TO THE PERINATAL PERIOD |
| | 7718 | OTHER TYPE OF INFECTION SPECIFIC TO THE PERINATAL PERIOD |
| | 7701 2 | PNEUMONIA DUE TO MECONIUM ASPIRATION |
| | 7718 0 | SEPTICEMIA (SEPSIS) OF NEWBORN |
| | 77181 | SEPTICEMIA (SEPSIS) OF NEWBORN |
| | 77182 | URINARY TRACT INFECTION OF NEWBORN |
| **Nosocomial infections** | V0991 | INFECTION WITH DRUG-RESISTANT MICROORGAN.,UNSPEC. DRUG RESISTANCE NOS, WITH MULTIPLE DRUG RESISTANCE |
| | V091 | INFECTION WITH MICROORGAN. RESISTANT TO CEPHALOSPORINS/B-LACTAM ANTIBIOTICS |
| | V0980 | INFECTION WITH MICROORGAN. RESISTANT TO OTHER SPEC. DRUGS, WITHOUT MENTION OF RESISTANCE TO MULTIPLE DRUGS |
| | 00845 | INTESTINAL INFEC. DUE TO CLOSTRIDIUM DIFFICILE |
| **Ophthalmic infections** | 37313 | ABSCESS OF EYELID |
| | 37300 | BLEPHARITIS, UNSPECIFIED |
| | 37230 | CONJUNCTIVITIS, UNSPECIFIED |
| | 37601 | ORBITAL CELLULITIS |
| | 376010 | PERIORBITAL CELLULITIS |
| **Orthopedic infections** | 73007 | ACUTE OSTEOMYELITIS INVOLVING ANKLE AND FOOT |
| | 73003 | ACUTE OSTEOMYELITIS INVOLVING FOREARM |
| | 73004 | ACUTE OSTEOMYELITIS INVOLVING HAND |
| | 73006 | ACUTE OSTEOMYELITIS INVOLVING LOWER LEG |
| | 73008 | ACUTE OSTEOMYELITIS INVOLVING OTHER SPECIFIED SITES |
| | 73005 | ACUTE OSTEOMYELITIS INVOLVING PELVIC REGION AND THIGH |
| | 73001 | ACUTE OSTEOMYELITIS INVOLVING SHOULDER REGION |
| | 73000 | ACUTE OSTEOMYELITIS, SITE UNSPECIFIED |
| | 71156 | ARTHROPATHY LOWER LEG (INCL.KNEE) WITH OTHER VIRAL DISEASES |
| | 71146 | ARTHROPATHY LOWER LEG(INCL.KNEE)WITH OTHER BACTERIAL DISEASES |
| | 71148 | ARTHROPATHY OTHER SPECIF.SITES,WITH OTHER BACTERIAL DISEASES |
| | 71145 | ARTHROPATHY PELVIC REGION/THIGH,WITH OTHER BACTERIAL DISEASES |

| | | |
|---|---|---|
| | 71141 | ARTHROPATHY SHOULDER REGION,WITH OTHER BACTERIAL DISEASES |
| | 7301 | CHRONIC OSTEOMYELITIS |
| | 73017 | CHRONIC OSTEOMYELITIS INVOLVING ANKLE AND FOOT |
| | 73013 | CHRONIC OSTEOMYELITIS INVOLVING FOREARM |
| | 73014 | CHRONIC OSTEOMYELITIS INVOLVING HAND |
| | 73016 | CHRONIC OSTEOMYELITIS INVOLVING LOWER LEG |
| | 73019 | CHRONIC OSTEOMYELITIS INVOLVING MULTIPLE SITES |
| | 73018 | CHRONIC OSTEOMYELITIS INVOLVING OTHER SPECIFIED SITES |
| | 73015 | CHRONIC OSTEOMYELITIS INVOLVING PELVIC REGION AND THIGH |
| | 73011 | CHRONIC OSTEOMYELITIS INVOLVING SHOULDER REGION |
| | 73012 | CHRONIC OSTEOMYELITIS INVOLVING UPPER ARM |
| | 73010 | CHRONIC OSTEOMYELITIS, SITE UNSPECIFIED |
| | 73089 | INFECT.INVOLV.BONE OF MULT.SITES,IN DISEASES CLASSIF.ELSEWHERE |
| | 03682 | MENINGOCOCCAL ARTHROPATHY |
| | 7110 | PYOGENIC ARTHRITIS |
| | 71107 | PYOGENIC ARTHRITIS INVOLVING ANKLE AND FOOT |
| | 71103 | PYOGENIC ARTHRITIS INVOLVING FOREARM |
| | 71104 | PYOGENIC ARTHRITIS INVOLVING HAND |
| | 71106 | PYOGENIC ARTHRITIS INVOLVING LOWER LEG (INCL.KNEE) |
| | 71109 | PYOGENIC ARTHRITIS INVOLVING MULTIPLE SITES |
| | 71105 | PYOGENIC ARTHRITIS INVOLVING PELVIC REGION AND THIGH |
| | 71101 | PYOGENIC ARTHRITIS INVOLVING SHOULDER REGION |
| | 71100 | PYOGENIC ARTHRITIS, SITE UNSPECIFIED |
| | 71191 | UNSPECIFIED INFECTIVE ARTHRITIS INVOLVING SHOULDER REGION |
| | 7302 | UNSPECIFIED OSTEOMYELITIS |
| | 73027 | UNSPECIFIED OSTEOMYELITIS INVOLVING ANKLE AND FOOT |
| | 73023 | UNSPECIFIED OSTEOMYELITIS INVOLVING FOREARM |
| | 73024 | UNSPECIFIED OSTEOMYELITIS INVOLVING HAND |
| | 73026 | UNSPECIFIED OSTEOMYELITIS INVOLVING LOWER LEG |
| | 73029 | UNSPECIFIED OSTEOMYELITIS INVOLVING MULTIPLE SITES |
| | 73028 | UNSPECIFIED OSTEOMYELITIS INVOLVING OTHER SPECIFIED SITES |
| | 73025 | UNSPECIFIED OSTEOMYELITIS INVOLVING PELVIC REGION AND THIGH |
| | 73021 | UNSPECIFIED OSTEOMYELITIS INVOLVING SHOULDER REGION |
| | 73022 | UNSPECIFIED OSTEOMYELITIS INVOLVING UPPER ARM |
| | 73020 | UNSPECIFIED OSTEOMYELITIS, SITE UNSPECIFIED |
| **Other infections** | 03283 | DIPHTHERITIC PERITONITIS |
| | 0312 | DISSEMINATED DISEASE DUE TO OTHER MYCOBACTERIA |
| | 0418 | OTHER SPEC.BACTERIAL INF;IN CONDIT.CLASS.ELSEWHERE,UNSP.SITE |
| | 04189 | OTHER SPECIFIED BACTERIA INFECTION |
| | 01485 | OTHER TB. INTESTINES, CONFIRMED HISTOLOGICALLY |
| | 01090 | PRIM. TB. INFEC., UNSP.TYPE, UNSP.EXAMINATION |
| | 0940 | TABES DORSALIS |
| | 01304 | TB. MENINGITIS, FOUND BY BACTERIAL CULTURE |
| | 01120 | TB. OF LUNG + CAVITATION, UNSP. EXAMINATION |
| | 01404 | TB. PERITONITIS, FOUND BY BACTERIAL CULTURE |

| | | |
|---|---|---|
| | 01311 | TUBERCULOMA OF MENINGES, BACT/HISTOL. EXAM. NOT DONE |
| | 0419 | UNSP. BACTERIAL INFECTION, UNSP. SITE |
| | 01194 | UNSP. PULMONARY TB., FOUND BY BACTERIAL CULTURE |
| | 01190 | UNSP. PULMONARY TB., UNSP. EXAMINATION |
| | 01394 | UNSP. TB. OF C.N.S. FOUND BY BACTERIAL CULTURE |
| | 0419 | UNSPEC.BACTERIAL INF;IN CONDIT. CLASSIF.ELSEWHERE,UNSP.SITE |
| | 0319 | UNSPECIFIED DISEASES DUE TO MYCOBACTERIA |
| | 0119 | UNSPECIFIED PULMONARY TUBERCULOSIS |
| **Respiratory infections** | 0796 | RESPIRATORY SYNCYTIAL VIRUS (RSV) |
| | 0793 | RHINOVIRUS INFECTION, UNSP. SITE |
| | 485 | BRONCHOPNEUMONIA, ORGANISM UNSPECIFIED |
| | 5100 | EMPYEMA WITH FISTULA |
| | 5109 | EMPYEMA WITHOUT MENTION OF FISTULA |
| | 486 | PNEUMONIA, ORGANISM UNSPECIFIED |
| | 5130 | ABSCESS OF LUNG |
| | 46619 | AC. BRONCHIOLITIS DUE TO OTHER INFECTIOUS ORGANISMS |
| | 46611 | AC. BRONCHIOLITIS DUE TO RESPIRATORY SYNCYTIAL VIRUS (RSV) |
| | 4661 | ACUTE BRONCHIOLITIS |
| | 4660 | ACUTE BRONCHITIS |
| | 466 | ACUTE BRONCHITIS AND BRONCHIOLITIS |
| | 46430 | ACUTE EPIGLOTTITIS WITHOUT MENTION OF OBSTRUCTION |
| | 4612 | ACUTE ETHMOIDAL SINUSITIS |
| | 4611 | ACUTE FRONTAL SINUSITIS |
| | 4640 | ACUTE LARYNGITIS |
| | 464 | ACUTE LARYNGITIS AND TRACHEITIS |
| | 46400 | ACUTE LARYNGITIS WITHOUT MENTION OF OBSTRUCTION |
| | 4650 | ACUTE LARYNGOPHARYNGITIS |
| | 46420 | ACUTE LARYNGOTRACHEITIS WITHOUT MENTION OF OBSTRUCTION |
| | 4610 | ACUTE MAXILLARY SINUSITIS |
| | 460 | ACUTE NASOPHARYNGITIS (COMMON COLD) |
| | 462 | ACUTE PHARYNGITIS |
| | 4619 | ACUTE SINUSITIS, UNSPECIFIED |
| | 4613 | ACUTE SPHENOIDAL SINUSITIS |
| | 463 | ACUTE TONSILLITIS |
| | 46410 | ACUTE TRACHEITIS WITHOUT MENTION OF OBSTRUCTION |
| | 465 | ACUTE UPPER RESPIRATORY INFECTIONS OF MULTIPLE OR UNSP.SITES |
| | 4658 | ACUTE UPPER RESPIRATORY INFECTIONS OF OTHER MULTIPLE SITES |
| | 4659 | ACUTE UPPER RESPIRATORY INFECTIONS OF UNSPECIFIED SITE |
| | 4829 | BACTERIAL PNEUMONIA, UNSPECIFIED |
| | 490 | BRONCHITIS, NOT SPECIFIED AS ACUTE OR CHRONIC |
| | 4732 | CHRONIC ETHMOIDAL SINUSITIS |
| | 4731 | CHRONIC FRONTAL SINUSITIS |
| | 4730 | CHRONIC MAXILLARY SINUSITIS |
| | 4720 | CHRONIC RHINITIS |
| | 4733 | CHRONIC SPHENOIDAL SINUSITIS |

| | | |
|---|---|---|
| | 4644 | CROUP |
| | 4880 | INFLUENZA DUE TO IDENTIFIED AVIAN INFLUENZA VIRUS |
| | 4881 | INFLUENZA DUE TO IDENTIFIED NOVEL H1N1 INFLUENZA VIRUS |
| | 4878 | INFLUENZA WITH OTHER MANIFESTATIONS |
| | 4871 | INFLUENZA WITH OTHER RESPIRATORY MANIFESTATIONS |
| | 4870 | INFLUENZA WITH PNEUMONIA |
| | 49121 | OBSTRUCTIVE CHR. BRONCHITIS WITH(ACUTE)EXACERBATION |
| | 4618 | OTHER ACUTE SINUSITIS |
| | 4738 | OTHER CHRONIC SINUSITIS |
| | 47822 | PARAPHARYNGEAL ABSCESS |
| | 475 | PERITONSILLAR ABSCESS |
| | 481 | PNEUMOCOCCAL PNEUMONIA |
| | 481 | PNEUMOCOCCAL PNEUMONIA (STREPTOCOCUUS PNEUMONIAE PNEUMONIA) |
| | 4831 | PNEUMONIA DEU TO CHLAMYDIA |
| | 4800 | PNEUMONIA DUE TO ADENOVIRUS |
| | 4822 | PNEUMONIA DUE TO HEMOPHILUS INFLUENZAE (H. INFLUENZAE) |
| | 4820 | PNEUMONIA DUE TO KLEBSIELLA PNEUMONIAE |
| | 4830 | PNEUMONIA DUE TO MYCOPLASMA PNEUMONIAE |
| | 483 | PNEUMONIA DUE TO OTHER SPECIFIED ORGANISM |
| | 4838 | PNEUMONIA DUE TO OTHER SPECIFIED ORGANISM |
| | 4808 | PNEUMONIA DUE TO OTHER VIRUS NOT ELSEWHERE CLASSIFIED |
| | 4802 | PNEUMONIA DUE TO PARAINFLUENZA VIRUS |
| | 4821 | PNEUMONIA DUE TO PSEUDOMONAS |
| | 4801 | PNEUMONIA DUE TO RESPIRATORY SYNCYTIAL VIRUS |
| | 48241 | PNEUMONIA DUE TO STAPHYLOCOCCUS AUREUS |
| | 4823 | PNEUMONIA DUE TO STREPTOCOCCUS |
| | 48231 | PNEUMONIA DUE TO STREPTOCOCCUS, GROUP A |
| | 48230 | PNEUMONIA DUE TO STREPTOCOCCUS, UNSPECIFIED |
| | 4841 | PNEUMONIA IN CYTOMEGALIC INCLUSION DISEASE |
| | 47824 | RETROPHARYNGEAL ABSCESS |
| | 0341 | SCARLET FEVER |
| | 0340 | STREPTOCOCCAL SORE THROAT |
| | 034 | STREPTOCOCCAL SORE THROAT AND SCARLET FEVER |
| | 46450 | SUPRAGLOTTITIS WITHOUT MENTION OF OBSTRUCTION |
| | 4739 | UNSPECIFIED SINUSITIS (CHRONIC) |
| | 480 | VIRAL PNEUMONIA |
| | 4809 | VIRAL PNEUMONIA, UNSPECIFIED |
| | 0331 | WHOOPING COUGH DUE TO BORDETELLA PARAPERTUSSIS |
| | 0330 | WHOOPING COUGH DUE TO BORDETELLA PERTUSSIS (B. PERTUSSIS) |
| | 0339 | WHOOPING COUGH, UNSPECIFIED ORGANISM |
| **Skin infections** | 1123 | CANDIDIASIS OF SKIN AND NAILS |
| | 0400 | GAS GANGRENE |
| | 6828 1 | ABSCESS OF SCALP |
| | 1120 | CANDIDIASIS OF MOUTH |

| | | |
|---|---|---|
| | 6805 | CARBUNCLE AND FURUNCLE OF BUTTOCK |
| | 6800 | CARBUNCLE AND FURUNCLE OF FACE |
| | 6806 | CARBUNCLE AND FURUNCLE OF LEG, EXCEPT FOOT |
| | 6801 | CARBUNCLE AND FURUNCLE OF NECK |
| | 6809 | CARBUNCLE AND FURUNCLE OF UNSPECIFIED SITE |
| | 6825 | CELLULITIS AND ABSCESS OF BUTTOCK |
| | 6820 | CELLULITIS AND ABSCESS OF FACE |
| | 6827 | CELLULITIS AND ABSCESS OF FOOT, EXCEPT TOES |
| | 6824 | CELLULITIS AND ABSCESS OF HAND, EXCEPT FINGERS AND THUMB |
| | 6826 | CELLULITIS AND ABSCESS OF LEG, EXCEPT FOOT |
| | 6821 | CELLULITIS AND ABSCESS OF NECK |
| | 6828 | CELLULITIS AND ABSCESS OF OTHER SPECIFIED SITES |
| | 6822 | CELLULITIS AND ABSCESS OF TRUNK |
| | 6819 | CELLULITIS AND ABSCESS OF UNSPECIFIED DIGIT |
| | 6829 | CELLULITIS AND ABSCESS OF UNSPECIFIED SITES |
| | 6823 | CELLULITIS AND ABSCESS OF UPPER ARM AND FOREARM |
| | 07811 | CONDYLOMA ACUMINATUM |
| | 1119 | DERMATOMYCOSIS, UNSPECIFIED |
| | 1104 | DERMATOPHYTOSIS OF FOOT |
| | 1103 | DERMATOPHYTOSIS OF GROIN AND PERIANAL AREA |
| | 1101 | DERMATOPHYTOSIS OF NAIL |
| | 1100 | DERMATOPHYTOSIS OF SCALP AND BEARD |
| | 1105 | DERMATOPHYTOSIS OF THE BODY |
| | 1109 | DERMATOPHYTOSIS OF UNSPECIFIED SITE |
| | 035 | ERYSIPELAS |
| | 684 | IMPETIGO |
| | 0859 | LEISHMANIASIS, UNSPECIFIED |
| | 0780 | MOLLUSCUM CONTAGIOSUM |
| | 72886 | NECROTIZING FASCIITIS |
| | 68609 | OTHER PYODERMA |
| | 1118 | OTHER SPECIFIED DERMATOMYCOSES |
| | 07819 | OTHER SPECIFIED VIRAL WARTS |
| | 6850 | PILONIDAL CYST WITH ABSCESS |
| | 6851 | PILONIDAL CYST WITHOUT MENTION OF ABSCESS |
| | 1110 | PITYRIASIS VERSICOLOR |
| | 07812 | PLANTAR WART |
| | 68600 | PYODERMA, UNSP. |
| | 68100 | UNSPECIFIED CELLULITIS AND ABSCESS OF FINGER |
| | 0781 | VIRAL WARTS |
| | 07810 | VIRAL WARTS, UNSPECIFIED |
| **Systemic febrile syndromes** | 0783 | CAT-SCRATCH DISEASE |
| | 04082 | TOXIC SHOCK SYNDROME |
| | 022 | ANTHRAX |
| | 08882 | BABESIOSIS |
| | 0239 | BRUCELLOSIS, UNSPECIFIED |

| | | |
|---|---|---|
| | 1000 | LEPTOSPIROSIS ICTEROHEMORRHAGICA |
| | 08881 | LYME DISEASE (ERYTHEMA CHRONICUM MIGRANS) |
| | 0846 | MALARIA, UNSPECIFIED |
| | 0810 | MURINE (ENDEMIC) TYPHUS |
| | 0838 | OTHER SPECIFIED RICKETTSIOSES |
| | 0209 | PLAGUE, UNSPECIFIED |
| | 0205 | PNEUMONIC PLAGUE, UNSPECIFIED |
| | 0830 | Q FEVER |
| | 0879 | RELAPSING FEVER, UNSPECIFIED |
| | 0839 | RICKETTSIOSIS, UNSPECIFIED |
| | 0820 | SPOTTED FEVERS |
| | 0269 | UNSPECIFIED RAT-BITE FEVER |
| **Urological infections** | 59010 | AC.PYELONEPHRITIS WITHOUT LESION OF RENAL MEDULLARY NECROSIS |
| | 5950 | ACUTE CYSTITIS |
| | 5901 | ACUTE PYELONEPHRITIS |
| | 0990 | CHANCROID |
| | 59581 | CYSTITIS CYSTICA |
| | 5959 | CYSTITIS, UNSPECIFIED |
| | 05410 | GENITAL HERPES, UNSPECIFIED |
| | 09840 | GONOCOCCAL CONJUNCTIVITIS (NEONATORUM) |
| | 0980 | GONOCOCCAL INFEC.,ACUTE, OF LOWER GENITOURINARY TRACT |
| | 09882 | GONOCOCCAL MENINGITIS |
| | 09886 | GONOCOCCAL PERITONITIS |
| | 60491 | ORCHITIS AND EPIDIDYMITIS IN DISEASES CLASSIFIED ELSEWHERE |
| | 60490 | ORCHITIS AND EPIDIDYMITIS, UNSPECIFIED |
| | 6040 | ORCHITIS, EPIDIDYMITIS, AND EPIDIDYMO-ORCHITIS, WITH ABSCESS |
| | 60499 | OTHER ORCHITIS,EPIDIDYMITIS,EPIDIDYMO-ORCHIT. WITHOUT ABSCESS |
| | 07988 | OTHER SPEC. CHLAMYDIAL INFECTION |
| | 59589 | OTHER SPECIFIED TYPES OF CYSTITIS |
| | 59789 | OTHER URETHRITIS |
| | 6019 | PROSTATITIS, UNSPECIFIED |
| | 59080 | PYELONEPHRITIS, UNSPECIFIED |
| | 5902 | RENAL AND PERINEPHRIC ABSCESS |
| | 07998 | UNSP. CHLAMYDIAL INFECTION |
| | 5970 | URETHRAL ABSCESS |
| | 59780 | URETHRITIS, UNSPECIFIED |
| | 5990 | URINARY TRACT INFECTION, SITE NOT SPECIFIED |
| **Viral infections** | 0790 | ADENOVIRUS INF.IN CONDITIONS CLASSIF.ELSEWHERE,UNSP.SITE |
| | 0790 | ADENOVIRUS INFECTION, UNSP. SITE |
| | 0527 | CHICKENPOX WITH OTHER SPECIFIED COMPLICATIONS |
| | 0528 | CHICKENPOX WITH UNSPECIFIED COMPLICATION |
| | 0792 | COXSACKIE VIRUS INFECTION, UNSP. SITE |
| | 0785 | CYTOMEGALIC INCLUSION DISEASE |
| | 0785 | CYTOMEGALOVIRAL DISEASE |
| | 0791 | ECHO VIRUS INFECTION, UNSP. SITE |

| | | |
|---|---|---|
| | 0540 | ECZEMA HERPETICUM |
| | 0774 | EPIDEMIC HEMORRHAGIC CONJUNCTIVITIS |
| | 0570 | ERYTHEMA INFECTIOSUM (FIFTH DISEASE) |
| | 0784 | FOOT AND MOUTH DISEASE |
| | 05311 | GENICULATE HERPES ZOSTER |
| | 0743 | HAND, FOOT, AND MOUTH DISEASE |
| | 05479 | HERPES SIMPLEX + OTHER SPEC. COMPLICATIONS |
| | 05440 | HERPES SIMPLEX + UNSP. OPHTHALMIC COMPLICATION |
| | 05441 | HERPES SIMPLEX DERMATITIS OF EYELID |
| | 05443 | HERPES SIMPLEX DISCIFORM KERATITIS |
| | 05449 | HERPES SIMPLEX WITH OTHER OPHTHALMIC COMPLICATIONS |
| | 0549 | HERPES SIMPLEX WITHOUT MENTION OF COMPLICATION |
| | 05319 | HERPES ZOSTER + OTHER NERVOUS SYSTEM COMPLICATIONS |
| | 05329 | HERPES ZOSTER + OTHER OPHTHALMIC COMPLICATIONS |
| | 05320 | HERPES ZOSTER DERMATITIS OF EYELID |
| | 0539 | HERPES ZOSTER WITHOUT MENTION OF COMPLICATION |
| | 0542 | HERPETIC GINGIVOSTOMATITIS |
| | 0546 | HERPETIC WHITLOW |
| | 075 | INFECTIOUS MONONUCLEOSIS |
| | 0559 | MEASLES WITHOUT MENTION OF COMPLICATION |
| | 0723 | MUMPS PANCREATITIS |
| | 0729 | MUMPS WITHOUT MENTION OF COMPLICATION |
| | 0773 | OTHER ADENOVIRAL CONJUNCTIVITIS |
| | 07989 | OTHER SPEC. VIRAL INFECTION |
| | 07889 | OTHER SPECIFIED DISEASES DUE TO VIRUSES |
| | 07889 | OTHER SPECIFIED DISEASES DUE TO VIRUSES AND CHLAMYDIAE |
| | 0578 | OTHER SPECIFIED VIRAL EXANTHEMATA |
| | 0798 | OTHER SPECIFIED VIRAL INFECTION CLASSIF.ELSEWHERE,UNSP.SITE |
| | 05810 | ROSEOLA INFANTUM, UNSPECIFIED |
| | 0569 | RUBELLA WITHOUT MENTION OF COMPLICATION |
| | 07799 | UNSP. DIS. OF CONJUNCTIVA DUE TO VIRUSES |
| | 0799 | UNSP. VIRAL & CHLANYDIAL INFECTION |
| | 07999 | UNSP. VIRAL INFECTION |
| | 07999 | UNSP. VIRAL INFECTION (ADDITIONAL CODE) |
| | 0799 | UNSP.VIRAL INFECT.IN CONDITIONS CLASSIF.ELSEWHERE,UNSP.SITE |
| | 0779 | UNSPEC.DISEASES OF CONJUNCTIVA DUE TO VIRUSES AND CHLAMYDIAE |
| | 0521 | VARICELLA (HEMORRHAGIC) PNEUMONITIS |
| | 0529 | VARICELLA WITHOUT MENTION OF COMPLICATION |
| | 0579 | VIRAL EXANTHEM, UNSPECIFIED |
| | 0701 | VIRAL HEPATITIS A WITHOUT HEPATIC COMA |
| | 0701 | VIRAL HEPATITIS A WITHOUT MENTION OF HEPATIC COMA |
